# Editorial: Extracellular Enzymes in Aquatic Environments: Exploring the Link Between Genomic Potential and Biogeochemical Consequences

**DOI:** 10.3389/fmicb.2019.01463

**Published:** 2019-06-26

**Authors:** Maria M. Sala, Judith Piontek, Sonja Endres, Anna M. Romani, Sonya Dyhrman, Andrew D. Steen

**Affiliations:** ^1^Department of Marine Biology and Oceanography, Institut de Ciències del Mar (ICM-CSIC), Barcelona, Spain; ^2^Biological Oceanography, Leibniz Institute for Baltic Sea Research Warnemünde, Rostock, Germany; ^3^Business Development and Technology Transfer Corporation of Schleswig-Holstein (WTSH), Kiel, Germany; ^4^Department of Environmental Sciences, Institute of Aquatic Ecology, University of Girona, Girona, Spain; ^5^Department of Earth and Environmental Sciences, Columbia University, New York, NY, United States; ^6^Lamont-Doherty Earth Observatory, Palisades, NY, United States; ^7^Departments of Microbiology and Earth and Planetary Sciences, University of Tennessee, Knoxville, TN, United States

**Keywords:** enzymes, microorganisms, aquatic environment, degradation, bacterial activity

Microbes drive the Earth's biogeochemical cycles, exerting profound control over the global cycling of carbon and other elements (Falkowski et al., 2008). In aquatic systems, the importance of microbial extracellular enzymes to the mobilization, transformation, and turnover of organic and inorganic compounds in aquatic environments has been proved since the 80's (Hoppe, 1983; Chróst, 1989) and was summarized in the book “Microbial enzymes in aquatic environments” (Chróst, 1991). Since then, the field has advanced considerably, with new observations, assay methods, and molecular-level studies (Arnosti et al., 2014) and the measurement of extracellular enzyme activities has become standard in many labs. We now have rates of enzymatic activities in a wide variety of freshwater and marine environments, from polar to tropical and from surface to deep ocean, and from isolates obtained even from extreme environments. Additionally, in recent years measurement of enzyme activities has become an important tool to assess the impact of anthropogenic changes on microbial communities and biogeochemical cycles, such as in the events of oil spills, acidification, or global warming (e.g., Piontek et al., 2010; Sala et al., 2016; Ziervogel et al., 2016; Freixa et al., 2017).

In this special issue, twelve articles highlight new findings on extracellular enzyme activities in aquatic environments, bringing together experimental and field studies conducted in marine and freshwater ecosystems as well as physiological, biochemical, and molecular studies on microbial communities or species isolated from those environments.

The first part of the volume is devoted to field studies, both in marine and freshwater ecosystems. To begin, the perspective article by Baltar advocates the need to go “beyond the living things” and study cell-free enzymatic activities to fully constrain the future and evolution of marine biogeochemical cycles. In marine environments, Hoarfrost and Arnosti show that the spectrum of substrates hydrolyzed in mesopelagic and deep waters of the Atlantic Ocean is positively related to the strength of stratification depth patterns, which may influence the efficiency of the biological carbon pump. Apart from their enzyme activities, knowing the types of bacteria that metabolize polymers can help make the critical connection between the taxonomic composition of microbial communities and their biogeochemical function. Liu et al. show, by using stable isotope probing, that a more diverse group of bacteria is involved in metabolizing peptides in normoxic surface water than in hypoxic bottom seawater from the Gulf of Mexico. In freshwaters, kinetic measurements of 5 substrates for exo- and endo-acting extracellular peptidases in 28 freshwater bodies in the Pocono Mountains (Mullen et al.) show variable ratios between aminopeptidases (APs) and trypsin, highlighting that measuring only Leu- AP activity may underestimate the total peptidolytic capacity in an environment. Spatial, but not seasonal, variability is also observed in a multi-season investigation in two North Carolina rivers examining the activities of extracellular enzymes used to hydrolyze polysaccharides and peptides (Bullock et al). Collectively, these studies expand our understanding of the role of microbial enzymes in the biogeochemistry of aquatic ecosystems.

Experimental approaches are increasingly used to tease apart the complex role aquatic microbial enzymes have on the biogeochemistry and functioning of ecosystems. Traving et al. in a mesocosm experiment observed the effect of increased loads of dissolved organic matter (DOM) in bacterioplankton community composition and a stimulation of protease activity. This suggests that parts of future elevated riverine DOM supply to the Baltic Sea will be efficiently mineralized by microbes and will have consequences in bacterioplankton and phytoplankton community composition and function. Indeed, organic matter released by phytoplankton fuels bacterial growth and the transformation of this DOM plays a role in the formation of chromophoric DOM which is ubiquitous in the ocean. Kinsey et al. investigated CDOM formation mediated by microbial processing of phytoplankton-derived aggregates. Measurements of hydrolytic enzyme rates along with the fluorescent properties of organic matter suggest that bacterial degradation activity changes the composition of chromophoric dissolved organic matter to more humic-like compounds. Another experimental study by Kamalanathan et al. reports on the role of bacterial extracellular enzymes during exposure to hydrocarbons and dispersant in mesocosm tanks and observed enhanced EPS (extracellular polymeric substances) production and extracellular enzyme activities in the oil amended treatment. These studies, which span both the field and laboratory, highlight the role of microbial enzymes in processing organic matter and the ramifications for diversity, resource remineralization, and community responses to anthropogenic perturbations.

Two of the papers in this issue address methodological concerns or improvements on enzyme activity measurements. Obayashi et al. use kinetic experiments to suggest improvements in material and methods for measurements of extracellular protease activities, and specifically highlight the relevance of using low protein binding materials. Also, Vrba et al. propose the fluorescence-labeled enzyme activity (FLEA) assay based in a novel substrate, ELF97 phosphate, that allows tagging extracellular phosphatase activity on single cells in an epifluorescence microscope and has shown to be useful in green algae cultures. This assay is shown being a strong tool for exploring plankton P metabolism.

The last part of the issue is focused on the characterization of new enzymes: a novel salt-tolerant esterase from deep-sea sediment of the South China Sea (Zhang et al.) and a new S8 serine protease from marine sedimentary *Photobacterium* sp. A5-7 (Li et al.).

The broad-range of articles presented in this topic deepens our understanding on the controls, activities, and biogeochemical consequences of microbial enzymes in aquatic environments. This body of work has, not only increased our knowledge, but also identified challenges and questions that remain open and should be addressed in the future.

## Author Contributions

All authors listed have made a substantial, direct and intellectual contribution to the work, and approved it for publication.

### Conflict of Interest Statement

The authors declare that the research was conducted in the absence of any commercial or financial relationships that could be construed as a potential conflict of interest.
